# Dysuria

**Published:** 1897-07

**Authors:** Lawrence M. Stanton

**Affiliations:** New York


### DYSURIA.

LAWRENCE M. STANTON, M. D., NEW YORK.

This case of painful micturition occurred in a young married woman.

Besides the pain experienced the urine contained a great quantity of mucus, but I do not remember that there was any pus present.

There was no fever, and there were no general symptoms, such as one would expect to find in a pronounced case of cystitis. But whether the case be called one of cystitis or dysuria, the homeopathic remedy cured it.

Urination was very frequent, and about one tablespoonful was voided each time, with severe pain just at the close of the act. The pain was "very low down," so probably at the mouth of the urethra.

In Allen's Boenninghausen's *Pocket Book*, under "Trouble at the Close of Micturition," are these remedies: Bry., Canth., Equisetum, Mez., Petrol., Phos., Sars., Sulph. Consulting the materia medica the symptoms under each of these remedies are as follows:

*Bry.* I have not been able to find any symptom occurring at the close of micturition under this remedy.

*Canth.* Burning at the end of scanty urination. Frequent micturition of scanty watery urine at first, with pain, and toward the close with cutting. Frequent urging, with scanty urine, and toward end of micturition pressing pain in base of uretha extending to orifice.

*Equisetum-hyemale.* Dysuria of women, with extreme and frequent urging to urinate, with severe pain, especially immediately after urine is voided.

*Mez.* Biting burning in forepart of urethra at close of micturition.

*Petrol.* Cutting in neck of bladder, at the beginning and close of micturition.

*Phos.* At close of micturition and after it, a smarting pain in glans.

*Sars.* Much pain at conclusion of passing water, almost unbearable.

*Sulph.* Cutting, as if the urine were acrid, at close of micturition and afterward.

On account of the pain being severe, but not of the cutting, burning, smarting variety, as under the above remedies, Sars. excepted, and because of the mucus in the urine, I decided upon Equisetum, and gave a dose of the 10M. (F.) potency.

Improvement began at once, and recovery was rapid and complete.

---

The following indications are found in the editor's notebook, and might be added to the foregoing:

*Sepia.* If the desire to urinate is not at once satisfied, a spasm occurs with trembling and shivering.

*Sulphuric-acid.* If the desire to urinate is not soon satisfied there is pain in the bladder.

*Causticum.* After urinating, smarting as if from salt in the pudendum.

*Phytolacca.* Pain in the region of the bladder before and during urination.

